# Supplementary figures and images for: Systematic Review of Available CAR-T Cell Trials around the World
Source: Cancers (Basel). 2022 May 27;14(11):2667. doi: 10.3390/cancers14112667 (PMC9179563; doi:10.3390/cancers14112667)

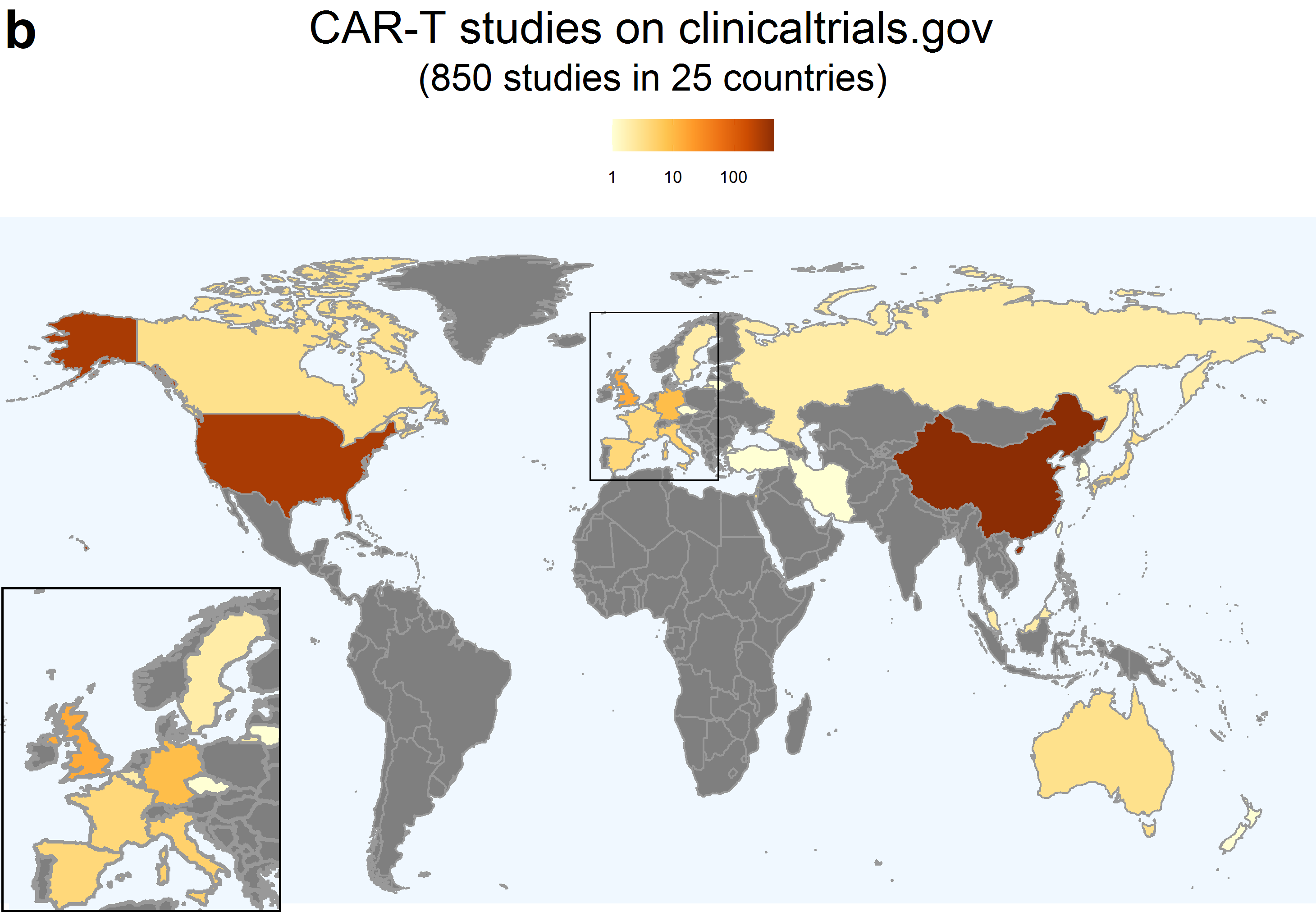

Supplement: Supplementary file 1 [file cancers-14-02667-s001.zip › LRCB_Supplementary Figure S1.png]

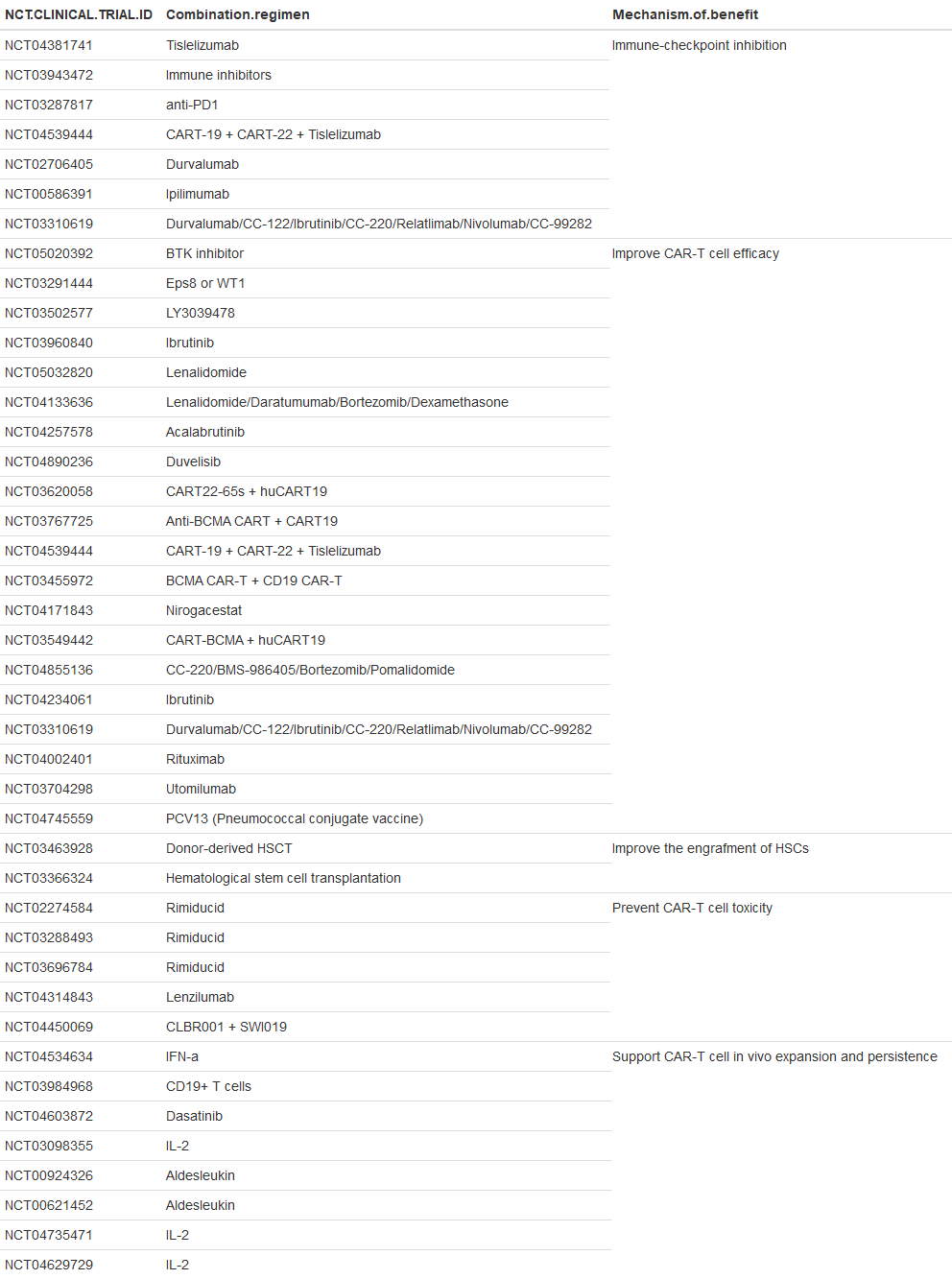

Supplement: Supplementary file 1 [file cancers-14-02667-s001.zip › Supplementary Table S2.png]

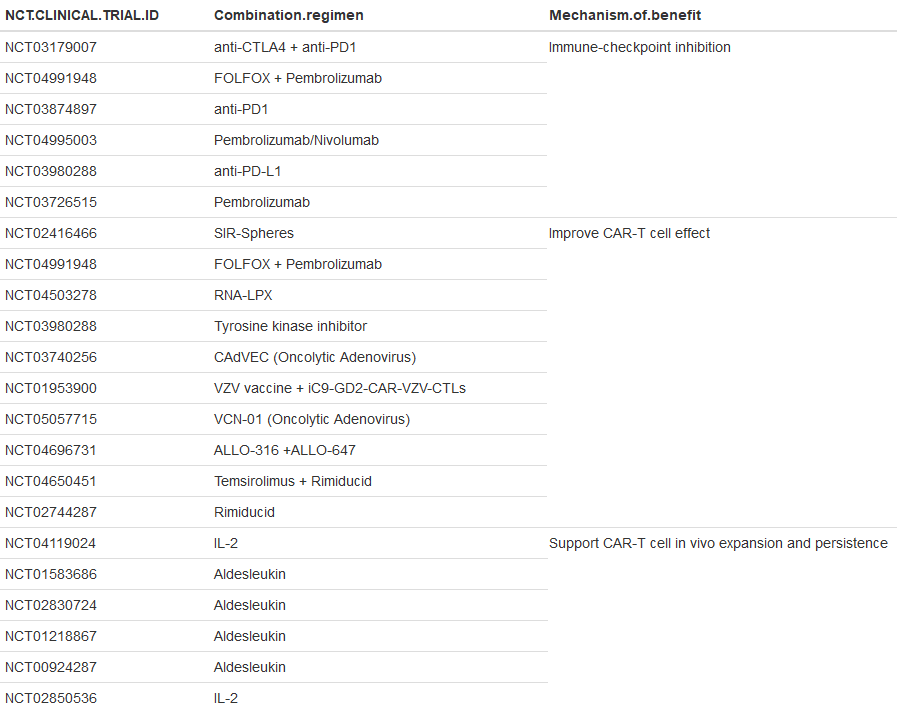

Supplement: Supplementary file 1 [file cancers-14-02667-s001.zip › Supplementary Table S3.png]
